# Transcriptome analysis of the tea oil camellia (*Camellia oleifera*) reveals candidate drought stress genes

**DOI:** 10.1371/journal.pone.0181835

**Published:** 2017-07-31

**Authors:** Bin Dong, Bin Wu, Wenhong Hong, Xiuping Li, Zhuo Li, Li Xue, Yongfang Huang

**Affiliations:** 1 South China Agricultural University, Guangzhou, Guangdong, China; 2 Guangdong Agriculture Industry Business Polytechnic, Guangzhou, Guangdong, China; 3 DRIGEN Company Limited, Shenzhen, Guangdong, China; 4 The Chinese University of Hong Kong, Hong Kong SAR, China; Dokuz Eylul Universitesi, TURKEY

## Abstract

**Background:**

The tea-oil camellia (*Camellia oleifera*) is the most important oil plant in southern China, and has a strong resistance to drought and barren soil. Understanding the molecular mechanisms of drought tolerance would greatly promote its cultivation and molecular breeding.

**Results:**

In total, we obtained 76,585 unigenes with an average length of 810 bp and an N50 of 1,092 bp. We mapped all the unigenes to the NCBI ‘nr’ (non-redundant), SwissProt, KEGG, and clusters of orthologous groups (COG) databases, where 52,531 (68.6%) unigenes were functionally annotated. According to the annotation, 46,171 (60.8%) unigenes belong to 338 KEGG pathways. We identified a series of unigenes that are related to the synthesis and regulation of abscisic acid (ABA), the activity of protective enzymes, vitamin B6 metabolism, the metabolism of osmolytes, and pathways related to the biosynthesis of secondary metabolites. After exposed to drought for 12 hours, the number of differentially-expressed genes (DEGs) between treated plants and control plants increased in the G4 cultivar, while there was no significant increase in the drought-tolerant C3 cultivar. DEGs associated with drought stress responsive pathways were identified by KEGG pathway enrichment analysis. Moreover, we found 789 DEGs related to transcription factors. Finally, according to the results of qRT-PCR, the expression levels of the 20 unigenes tested were consistent with the results of next-generation sequencing.

**Conclusions:**

In the present study, we identified a large set of cDNA unigenes from *C*. *oleifera* annotated using public databases. Further studies of DEGs involved in metabolic pathways related to drought stress and transcription will facilitate the discovery of novel genes involved in resistance to drought stress in this commercially important plant.

## Introduction

*Camellia oleifera*, an important member of the family Theaceae, is generally considered to be one of the four major woody oil trees in the world, together with the oil palm, olive, and coconut[1]. Tea oil, which is extracted from the seeds of *C*. *oleifera*, has been used as a high-grade cooking oil in China for hundreds of years. Because of its similar chemical composition to olive oil, tea oil has a reputation as the ‘eastern olive oil’. It is rich in several fatty acids, including palmitic, stearic, linoleic, oleic, and linolenic acids[2]. Most of these fatty acids have a significant effect on reducing the risk of cardiovascular disease, enhancing immunity, lowering cholesterol levels, preventing and treating hypertension, and protecting against certain cancers[3]. According to previous studies, all parts of *C*. *oleifera* are valuable, and its byproducts also have great economic values in agriculture, industry, and medicine[4–7]. Besides its economic value, *C*. *oleifera* has also demonstrated a role in water and soil conservation, biological fireproofing, and the improvement of ecological environments[8]. Currently, China is faced with an urgent demand to increase the percentage of self-sufficiency in cooking oil production. With its strong resistance to drought and barren soil in hilly regions, *C*. *oleifera* has gradually become the most important woody oil tree in China, where its cultivated area has been increased to more than 3.7 million hectares[9].

It is well known that drought is the most significant environmental stress in agriculture on a global scale [10]. Although *C*. *oleifera* is considered to be a drought-resistant tree, the serious water shortage in arid mountains or hilly lands render these areas extreme environments, and is considered to be the major obstacle to *C*. *oleifera* growth. In *C*. *oleifera*, drought can cause wilting, decreases in photosynthesis, oil contents, flower buds, and fruits setting, and even the death of drought-sensitive cultivars[11]. As the annual dry season in southern China (July to September) is always concomitant with high temperature, the recorded soil moisture has decreased significantly since 1950 in these areas[12].

In reality, plants developed a myriad of mechanisms to adapt or survive when exposed to drought stress, which could involve a series of molecular, biochemical, physiological, and morphological changes underlying the response of plants to water deprivation[13]. These strategies include reductions in growth rates and crop yields[14], enhancing root systems and root-shoot ratios to imbibe moisture[15], regulating the closure of stomata[16], disrupting photosynthetic pigments[17], activating respiration[18], accumulating compatible solutes and protective proteins[19, 20], and increasing the level of antioxidants[21, 22]. At the molecular level, the aforementioned changes will activate a range of responses, and most notably in drought-related genes that induce or inhibit gene expression. These changes also trigger the synthesis of specific biomolecules that cause a series of complex physiological and metamorphic alterations[23–26]. It has been suggested that stress-responsive genes can be more or less classified into two major categories based on their regulator molecules, the functions of their products, or their effector molecules[27–29]. The first group of molecules plays a direct role in abiotic stress tolerance, protect cells against damage, and sustain cell viability. These include enzymes involved in osmolyte biosynthesis, water channel proteins, sugar and proline transporters, detoxification enzymes, antioxidant proteins, chaperones, osmotin, antifreeze proteins, late embryogenesis abundant (LEA) proteins, and various proteases[30–32]. The second group is composed of regulatory proteins, including transcription factors (TFs), protein phosphatases and protein kinases, and other signaling molecules such as calmodulin-binding protein[33–35].

With the development of modern breeding technology, a number of elite cultivars of *C*. *oleifera* have thus far been authorized and released for tea oil production[36]. Several high-quality cultivars have various significant traits that are responsible for high-yield, high-oil, rapid growth, biotic and abiotic stress resistance, and so on. Understanding the genetic diversity and relatedness between various germplasm resources is critical for breeding new cultivars with more super-quality traits. In recent years, molecular markers have been frequently used in plant breeding, although this technology has limitations in the accuracy of localizing the target functional genes[37, 38]. The lack of genomic information has been an obstacle in the exploration of the molecular mechanisms of abioticstress in plants; however, with the advantage of RNA-sequencing (RNA-Seq), it is possible to analyze the transcriptomes of non-model plants transcriptome on a genome-wide scale[39–41]. This technique has been confirmed as an effective and feasible method to study drought resistance, and a suite of other traits, in a large number of non-model plants. With this technology, drought-tolerant genes have been successfully identified in a wide array of plants, such as *Populus trichocarpa*[42], *Gmelina arborea*[43], *Camellia sinensis*[44], *Hordeum vuglare* var. *nudum*[45], *Glycine max*[46], *Phormium tenax*[47] and *Pyrus betulaefolia*[48]. However, as a non-model species, little information regarding drought-related genes is available for *C*. *oleifera*, owing to the lack of genomic and transcriptomic studies[2, 49].

Thus, the present study aimed to identify cDNA unigenes from two *C*. *oleifera* cultivars via transcriptome analysis. To the best of our knowledge, this will be the first transcriptome sequencing of this non-model species under conditions of drought stress using NGS. We sequenced control samples and two *C*. *oleifera* cultivars that had been continuously treated with drought stress, and obtained vast numbers of unigenes annotated to public databases. We also used RNA-Seq data to compare the gene expression patterns of the two *C*. *oleifera* cultivars under conditions of drought stress. The resulting gene expression profiles will help us to better understand the mechanisms of drought resistance in *C*. *oleifera*.

## Results

### Illumina sequencing and read assembly

The 8 libraries were sequenced using the Illumina Hiseq 2500 platform. The total clean nucleotides generated were greater than 3 Gb for each sample, with at least 15 million raw reads per sample. Low-quality bases, including adapter sequences, were eliminated from the data used in subsequent analyses.

**Fig 1:**
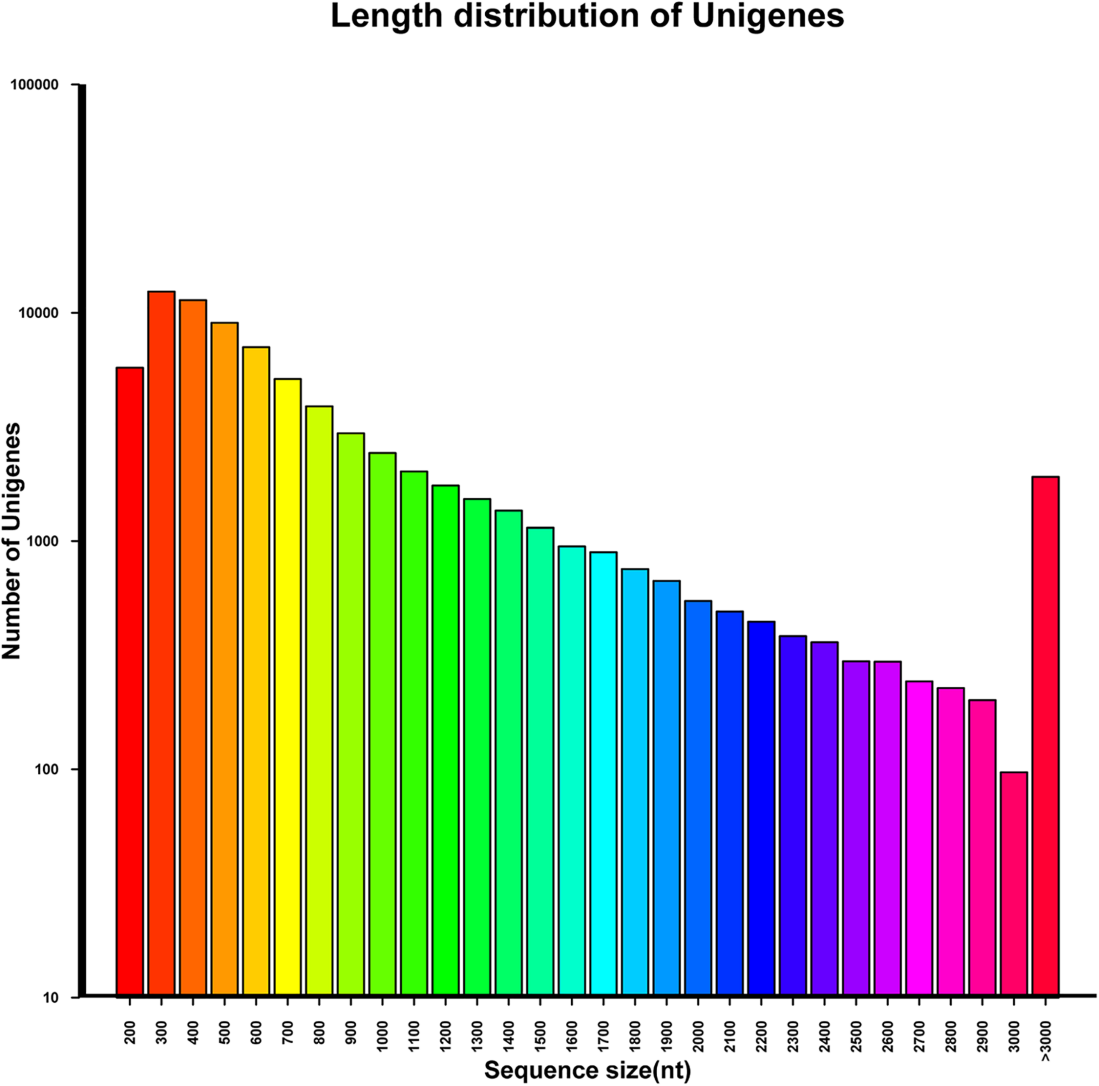
Length distribution of unigenes in *Camellia oleifera*.

Based on our bioinformatics analysis, we obtained 130 million clean reads from 8 samples. The clean reads were 40 Gb in length, and the Q20 percentage (which represents an error rate of 1 in 100, with a corresponding base calling accuracy of 99%) was 98% for each sample. Clean reads were submitted to the National Center for Biotechnology Information (NCBI) and can be downloaded from the Short Read Archive using the accession number SRP094080. CLC, a short-read assembling program, was used to implement *de novo* transcriptome assembly, and generated a total of 110,644 contigs. The average contig size of this assembly was 748 bp, while the N50 was 530 bp. We then used the TIGR Gene Indices clustering tool, TGICL, to connect the contigs and generated 76,585 unigenes (S1 Fig). The sequencing depth varied from 1to 55,230-fold with an average of 53-fold. The unigenes were 62,038,440 bp, 810 bp, and 1,092 bp in total length, mean length, and N50, respectively. All unigenes were longer than 200 bp (Fig 1).

### Functional annotation and classification

We subsequently aligned the 76,585 unigenes against protein databases using the basic local alignment search tool (BLAST), including nr (non-redundant protein sequences in NCBI), SwissProt, KEGG (Kyoto Encyclopedia of Genes and Genomes database), and COG (clusters of orthologous groups).

We found homologous sequences in the aforementioned databases (in at least one of the databases) for 52,531 (68.6%) unigenes, with a total of 46,602 (60.1%), 3,369 (4.4%), and 46,171 (33.2%) unigenes detected in the nr, SwissProt, and KEGG databases, respectively. Of these, there were 3,365 (4.4%) unigenes which had homologous sequences in all three databases, while a total of 15,954 (31.4%) unigenes were not identified in any of the aforementioned databases (Table 1).

**Table 1:** Functional annotation of the *Camellia oleifera* transcriptome.

Categories	Sequences(n)	Frequency(%)
All assembled unigenes	76,585	100
Annotated in SwissProt	3,369	4.40
Annotated in trembl	5,243	6.85
Annotated in tepep	5,927	7.74
Annotated in ipr	11,558	15.09
Annotated in GO	13,398	17.49
Annotated in COG	19,918	26.01
Annotated in KEGG	46,171	60.29
Annotated in nr	46,602	60.85
Annotated in nt	44,125	57.62
All Annotated unigenes	52,531	68.59

**Fig 2:**
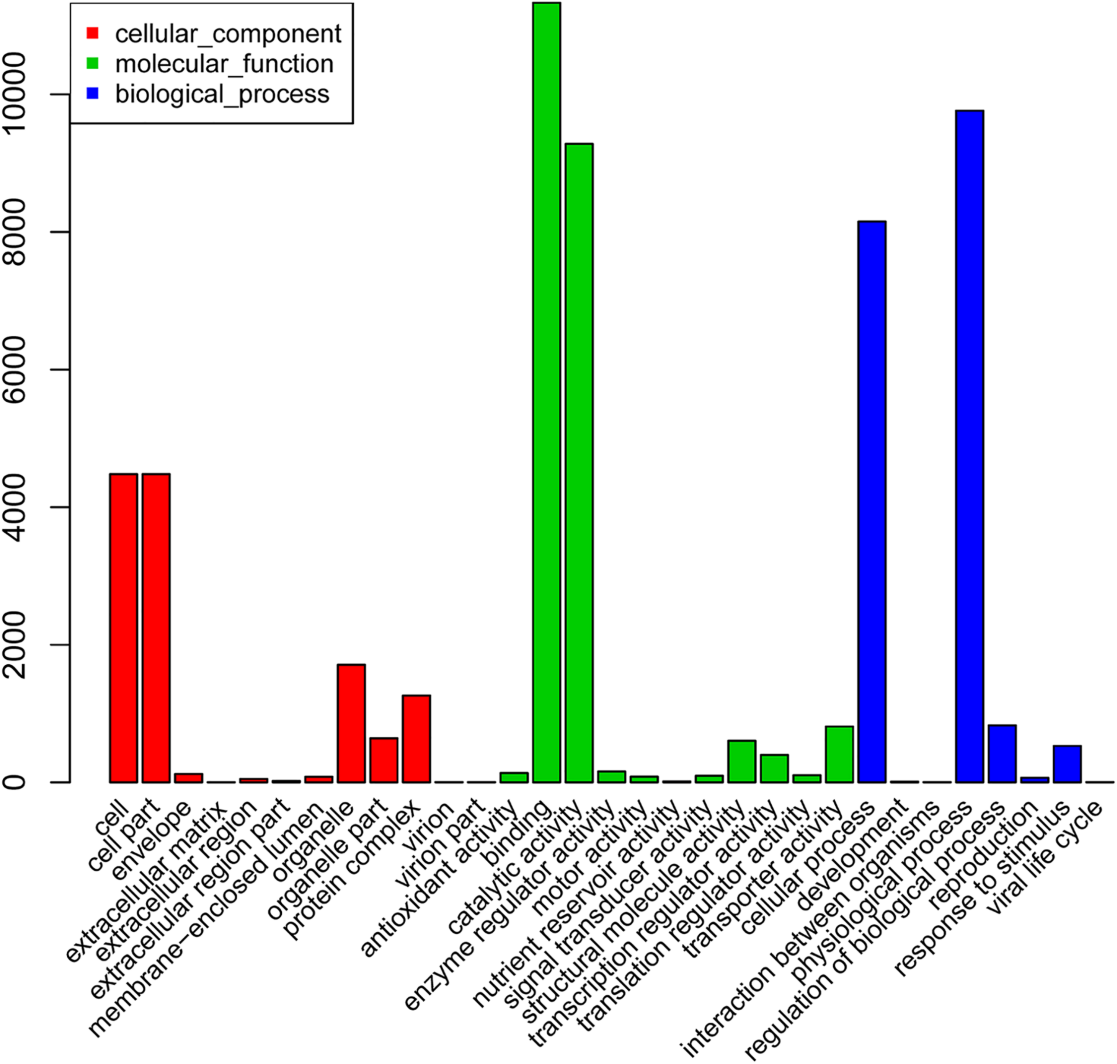
Distribution of gene ontology (GO) function in *Camellia oleifera*. The y-axis indicates the number of unigenes.

The GO classification system was applied to classify the possible functions of the assembled unigenes. In total, 19,412 (25.3%) unigenes were annotated with at least one GO term (Fig 2). According to the GO classification system, we grouped the unigenes into three main categories: biological process, cellular component, and molecular function. The biological process category contained 11,714 (15.3%) unigenes, which were annotated with 485 GO terms. The cellular components category contained 4,604 (6.0%) unigenes, which were annotated with 156 GO terms. Finally, the molecular function category contained 17,541 (22.9%) unigenes, which were annotated with 657 GO terms.

The top three largest categories in biological process were “physiological process” (9,762), “cellular process” (8,152), and “regulation of biological process” (829). “Cell” (4,481), “cell part” (4,481), and “organelle” (1,709) were the top three largest categories in the cellular component category. In the molecular function group, the top three largest categories were “binding” (11,332), “catalytic activity” (9,282), and “transporter activity” (813).

**Fig 3:**
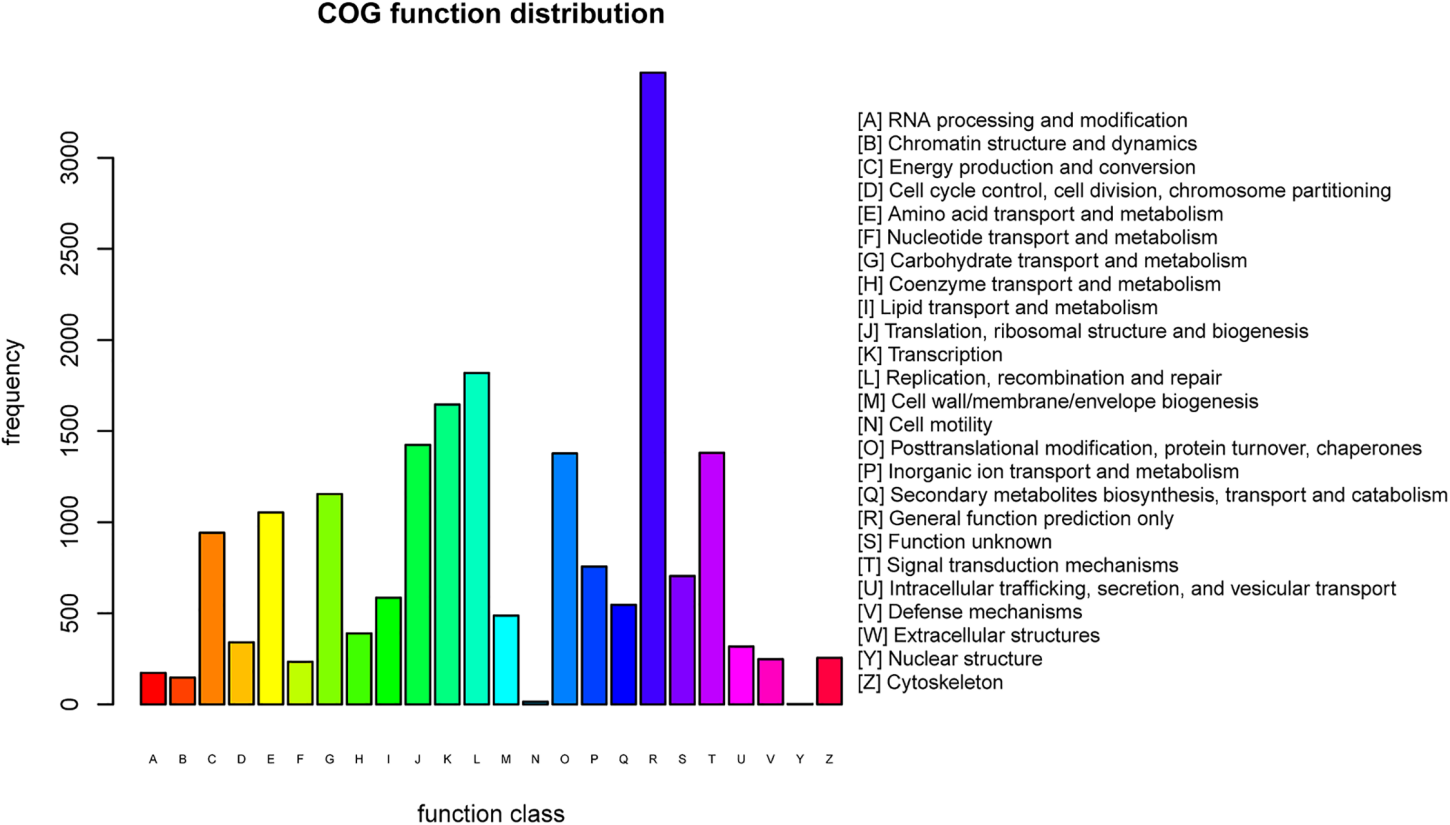
Clusters of orthologous groups (COG) classification of differentially expressed genes in *Camellia oleifera*.

To check the integrity of our transcriptome library, and to assess the effectiveness of the annotation process, all the unigenes were aligned to the COG database and 19,918 (26.0%) of them were identified to have homozygous sequences in COG. We then grouped these unigenes into 24 functional categories by classifying their possible functions (Fig 3).

Of the unigenes annotated by COG, The “general function” prediction was the largest category (3,467 of 19,918 unigenes, approx. 17.4%); followed by “replication, recombination, and repair” (1,819 unigenes, approx. 9.1%); transcription (1,646, 8.3%);“translation, ribosomal structure, and biogenesis” (1,424, 7.1%), and “signal transduction mechanisms” (1,380, 6.9%). The categories “chromatin structure and dynamics” (147, 0.74%), “cell motility” (15, 0.075%), and “nuclear structure” (3, 0.015%) contained the fewest genes. Finally, 704 (3.5%) unigenes were grouped into the category “function unknown”.

**Fig 4:**
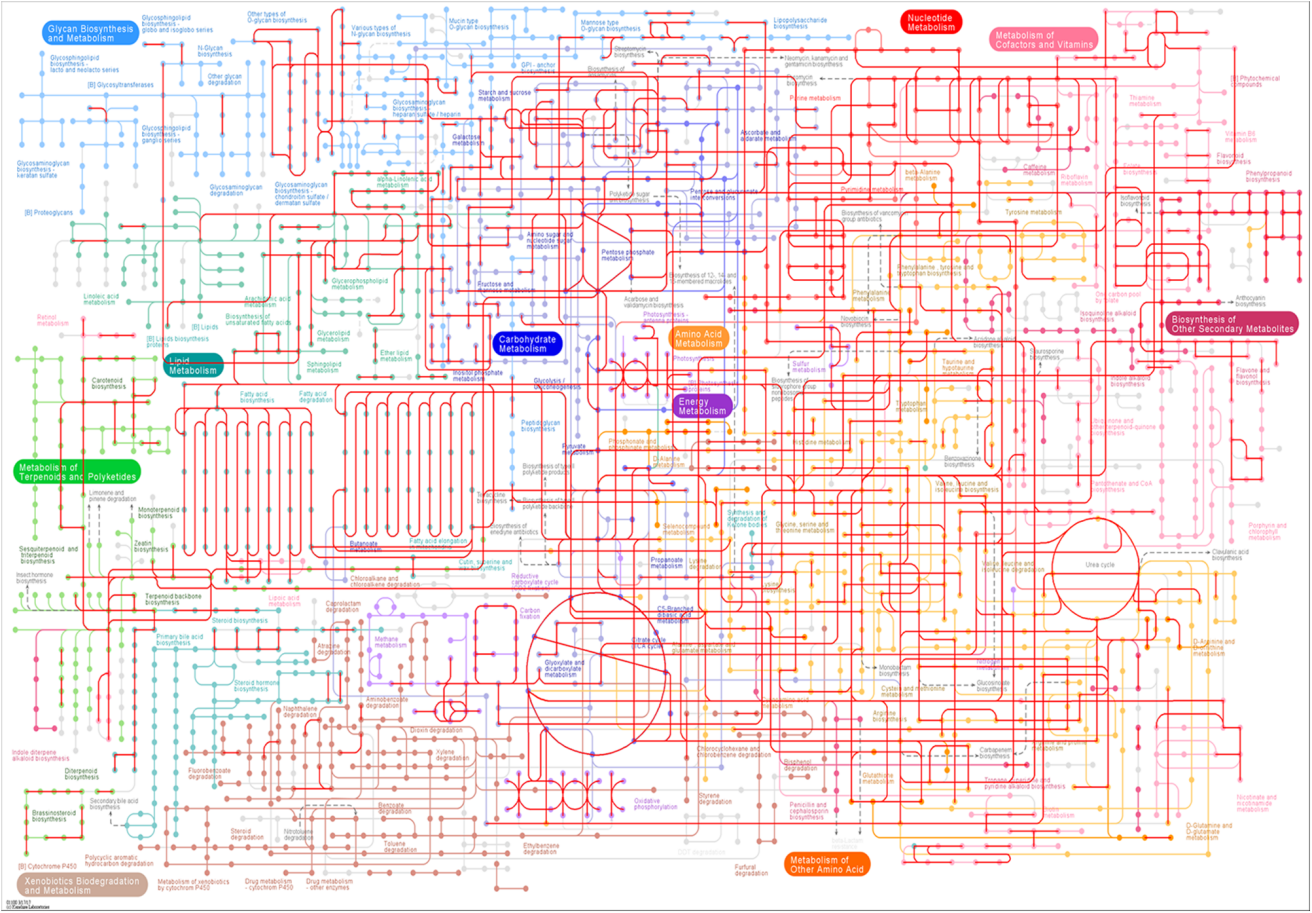
Global metabolic pathway in *Camellia oleifera*.

To further investigate gene products during metabolic processes, and determine their functions in cellular processes, we aligned the 76,585 unigenes to the KEGG database. A total of 46,171 (60.8%) unigenes had homologous sequences with 31,847 members that belong to 338 KEGG pathways. Of the 46,171 unigenes, 3,664 were involved in metabolic pathways, 1,875 were related to the biosynthesis of secondary metabolites, 651 to the ribosome, 569 to the biosynthesis of amino acids, and 374 to plant hormone signal transduction. To assess the global metabolic pathway, we mapped these unigenes on the Map01100 KEGG pathway (Fig 4). The majority of metabolic pathways were mapped, especially those involved in energy metabolism.

### Prediction of protein-coding regions

Based on the functional annotation, a total of 46,440 coding sequences (CDSs) were extracted from unigene sequences and translated into peptide sequences. The length distribution of the CDSs is shown in S2 Fig.

### Gene expression

**Fig 5:**
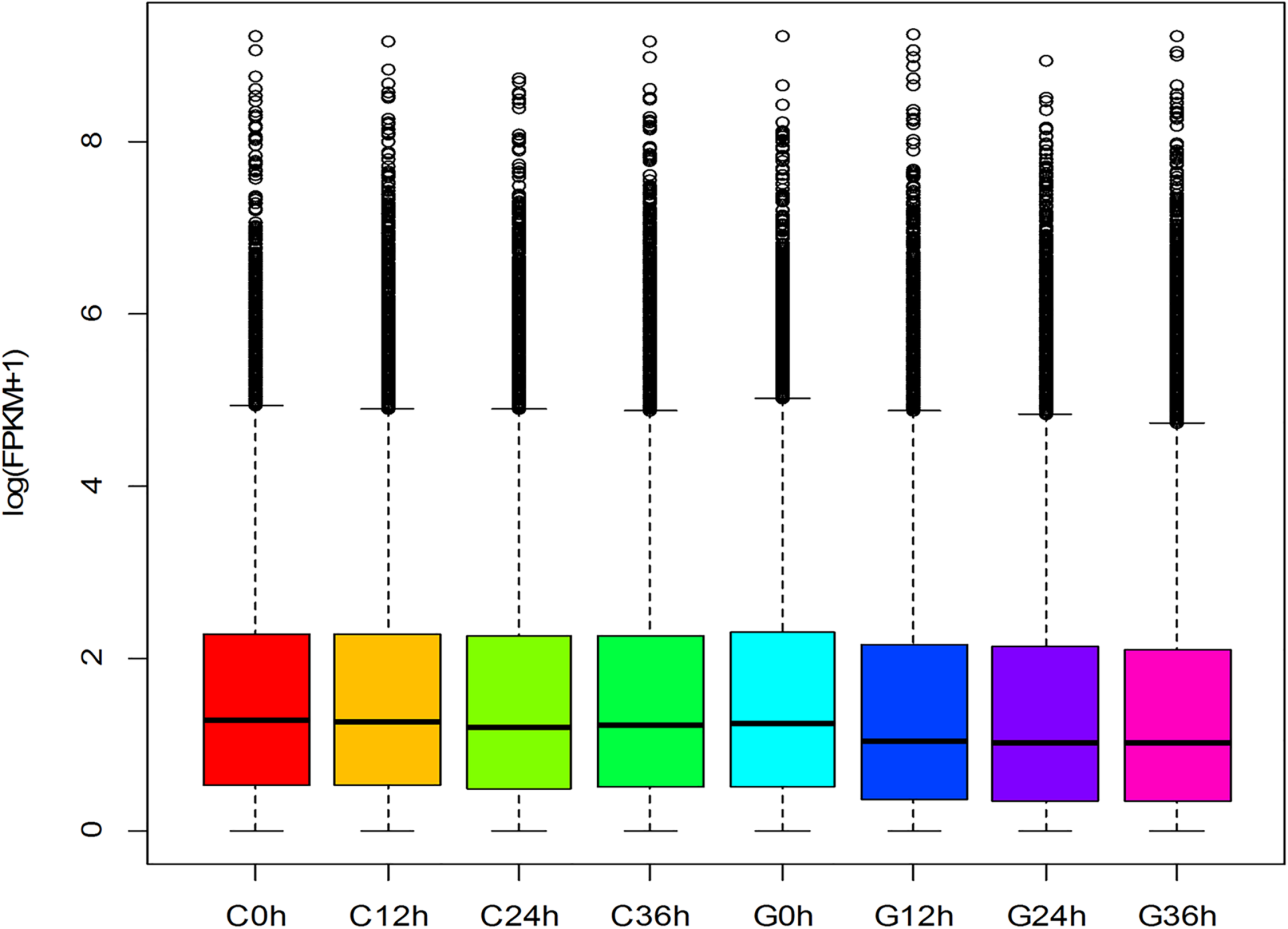
Distribution of gene expression. The distribution of gene expression in *Camellia oleifera*.

**Fig 6:**
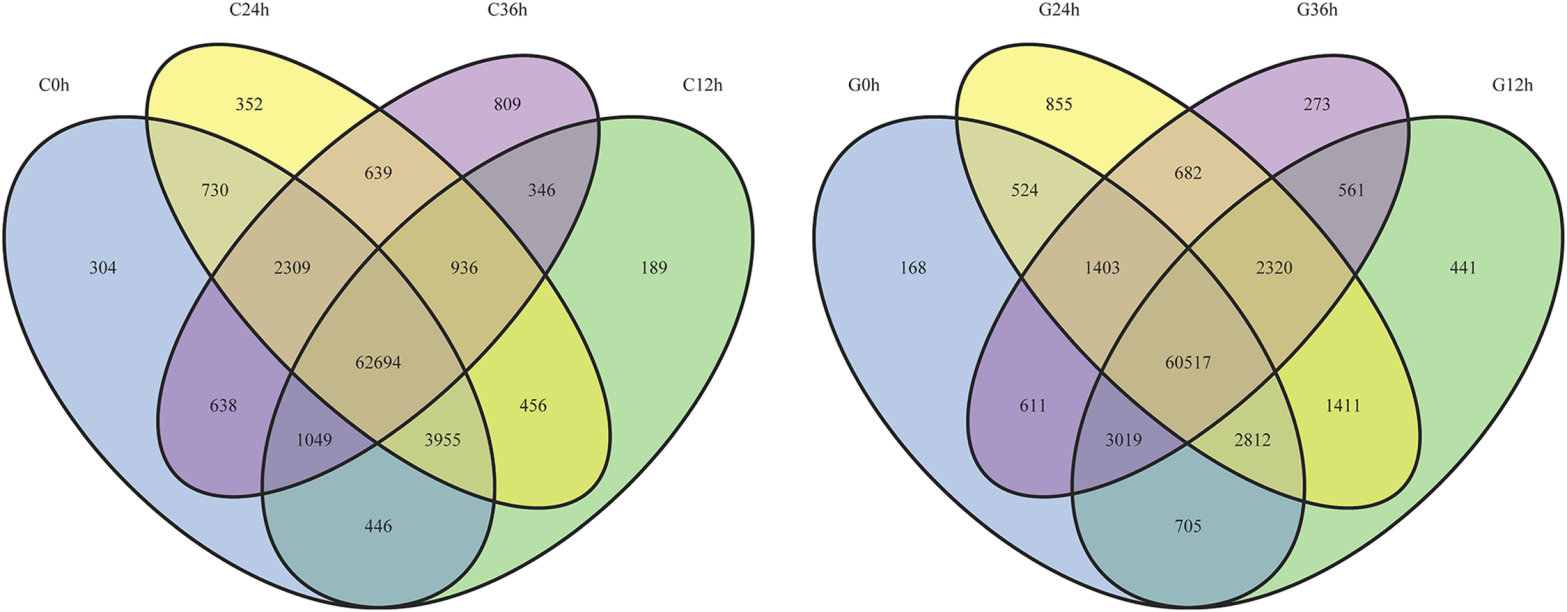
Genes from the C3 and G4 cultivars of *Camellia oleifera* showing treatment-specific expression during exposure to conditions of drought. Venn diagram showing the numbers of unigenes expressed as a result of exposure to conditions of drought over time (in C3 and G4 cultivars). The number of expressed genes shared between the four treatments is represented by overlapping circles.

The levels of expression of the unigenes from the 8 samples were calculated and compared by using the FPKM method (fragments per kb per million fragments). Fig 5 shows the distribution of gene expression of all unigenes. As a result, we observed that a large number of genes were specifically expressed in response to each treatment (Fig 6). The genes specifically expressed in response to exposure to drought stress are likely to be involved in the adaptation to this stress.

### Analysis of differential expression

**Fig 7:**
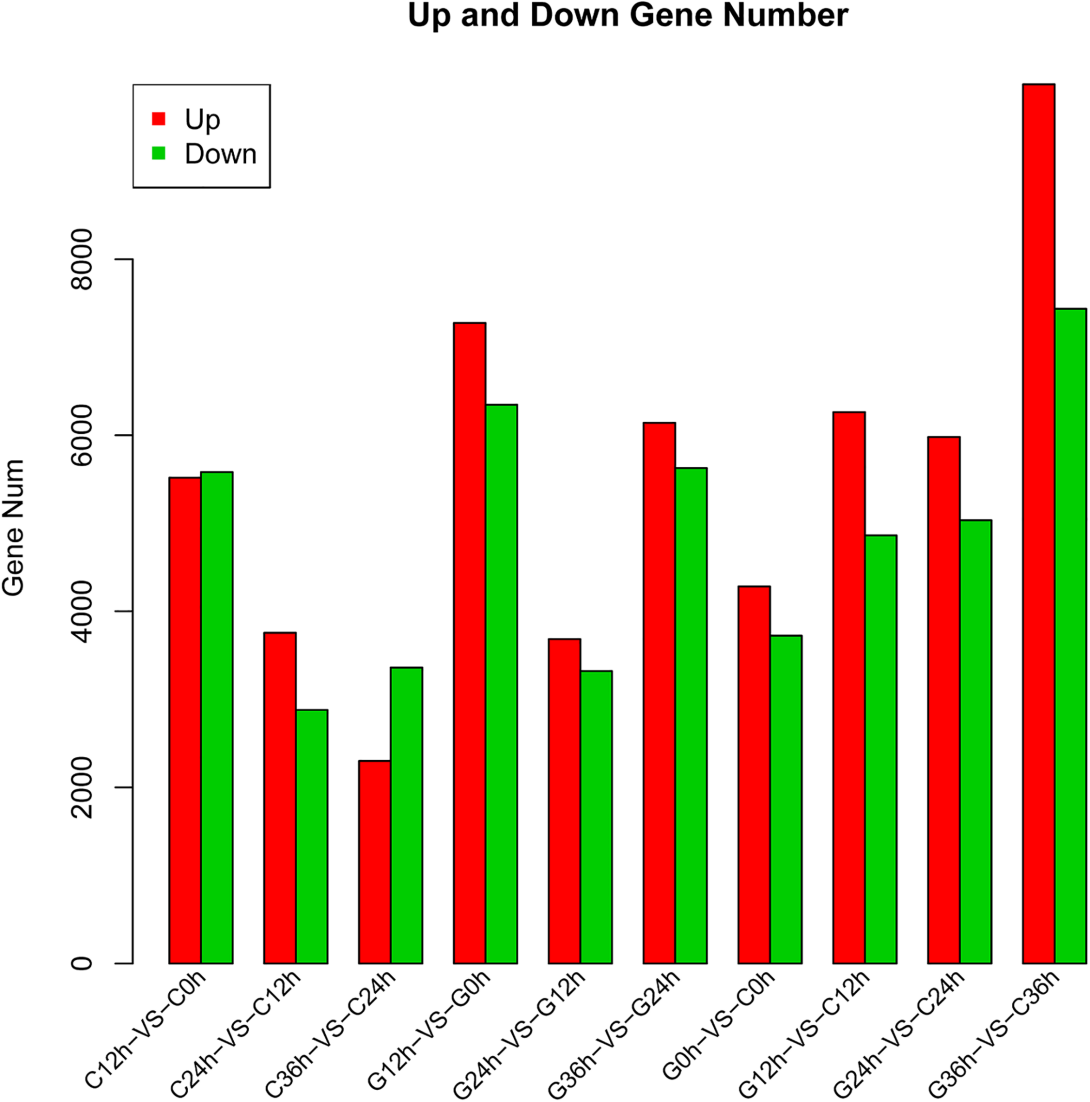
Number of differentially expressed genes in two *Camellia oleifera* cultivars.

In the FPKM analysis, we chose ten sample pairs, which consisted of a comparison between different treatment durations (0–12 h, 12–24 h, and 24–36 h) and a comparison between different cultivars (C3 vs G4). A gene was considered to be differentially expressed if the false discovery rate (FDR) was ≤ 0.001 and ratios were ≥ 2 (S1 Table). The distribution of up-regulated and down-regulated unigenes is shown in Fig 7.

When samples were not treated by drought stress, the number of differentially expressed unigenes (DEGs) was at its lowest level. Subsequently, this number increased slightly when plants were exposed to drought conditions for 24 hours. Although the differences between C3 24 h vs G4 24 h and C3 12 h vs G4 12 h were not significant, the number of up-regulated and down-regulated unigenes increased dramatically between the C3 and G4 cultivars after 36 hours of exposure to drought stress. Thus, is likely that the degree of drought-resistance differed between the two cultivars when treated for 36 hours.

In the C3 (drought-resistant) cultivar, the number of DEGs increased dramatically when exposed to drought conditions for 12 hours, and started to decline as the duration of exposure increased. There was slight increase in the number of up-regulated genes after 36 hours of drought stress in comparison to C3 24 h. In the G4 cultivar (drought-sensitive), the number of DEGs reached its peak after 12 hours of exposure to drought conditions, and started to decline as the duration of drought increased. However, the number of DEGs, both up-regulated and down-regulated, rebounded when the G4 cultivar was treated for 36 hours.

When exposed to conditions of drought for 12 hours, the C3 cultivar had 5,517 up-regulated unigenes and the G4 cultivar had 7,276, while 3,049 unigenes were shared between the two cultivars. The C3 cultivar had 5,581 down-regulated unigenes and the G4 cultivar had 6,346, while 2,536 unigenes were shared between the two cultivars.

**Fig 8:**
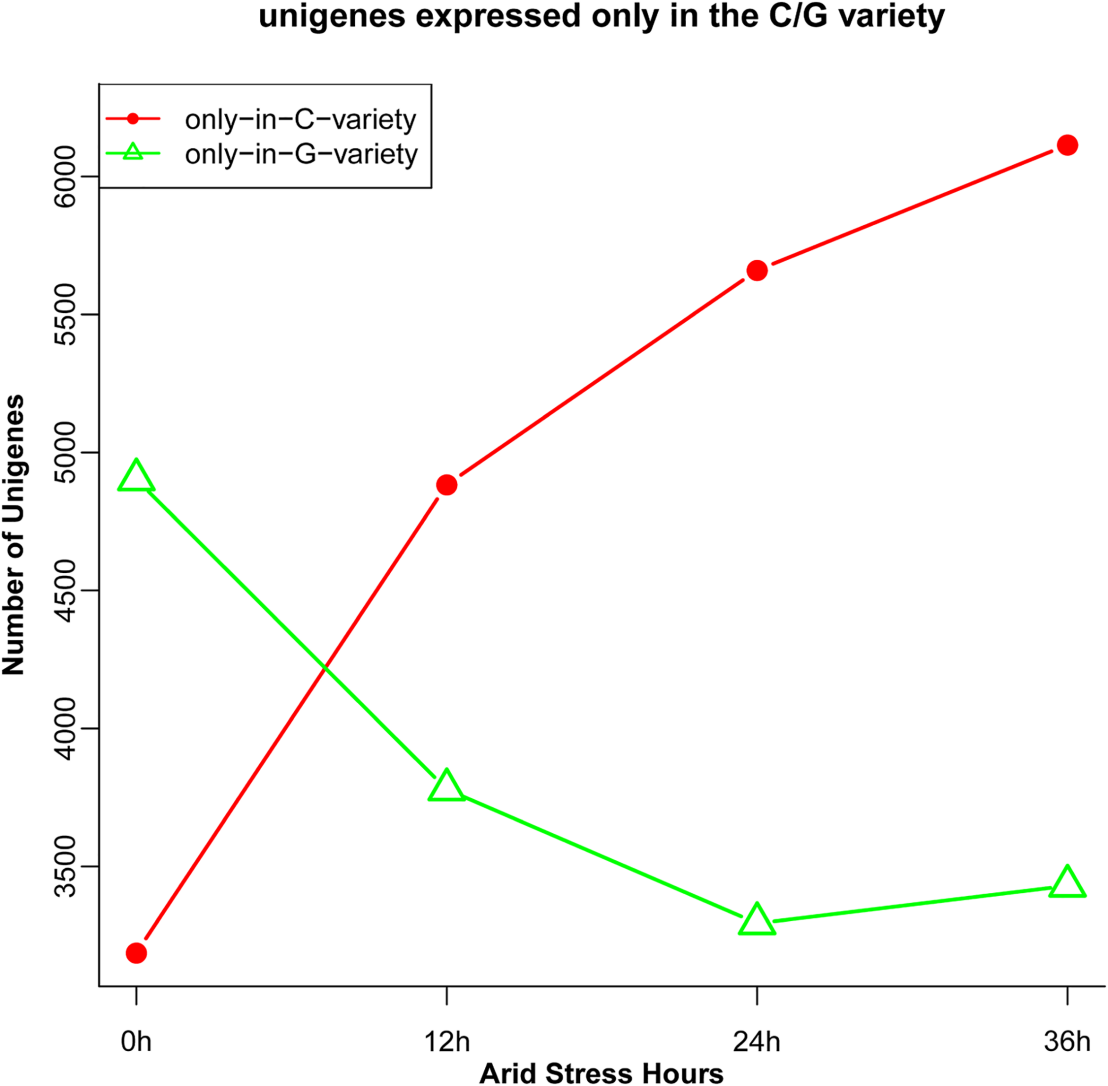
Number of differently expressed genes specific to the C3 and G4 cultivars of *Camellia oleifera*.

We also investigated unigenes that were only expressed in the C3 or G4 cultivar (Fig 8). As the exposure to stress continued, the number of unigenes that were expressed only in the C3 cultivar, continued to increase, while the number of unigenes expressed only in the G4 cultivar decreased.

DEGs from each comparison were then subjected to GO enrichment analysis. Using a hypergeometric test, we identified significantly enriched GO terms in DEGs in comparison to the genomic background. GO terms with a corrected *p* value of ≤0.05 were defined as a significantly enriched term for the DEGs. When exposed to drought stress for 12 hours, the DEGs between the C3 and G4 cultivar were significantly enriched in the processes of the biosynthesis of soluble sugars and proteins, and the processes of microtubule cytoskeleton organization. The results of the GO enrichment analysis are shown in S2 Table.

Based on the metabolic pathways enrichment analysis, we identified the primary biochemical pathways and signal transduction pathways associated with the DEGs. These DEGs were enriched in 39 metabolic pathways, including the biosynthesis of secondary metabolites, vitamin B6 metabolism, the metabolism of carbohydrates, and the photosynthesis pathway (S3 Table).

### Principle component analysis

**Fig 9:**
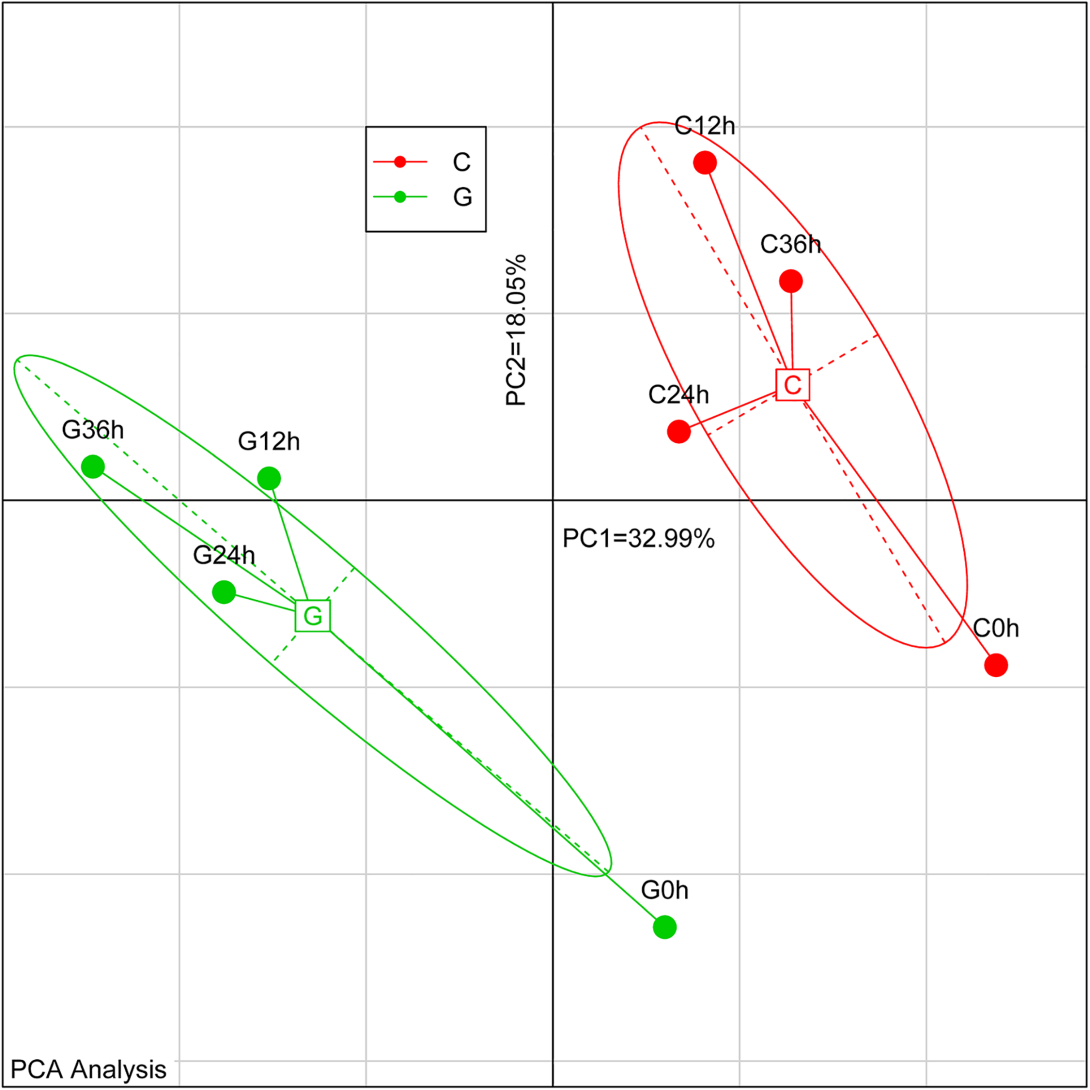
Principle component analysis (PCA) based on FPKM. PC1 and PC2 accounted for 32.9% and 18.05% of the principle components, respectively.

To further evaluate the difference in the levels of gene expression between the two cultivars, as well as between different time periods of drought exposure, we performed a principle component analysis of the 8 samples based on the FPKM values of all unigenes (Fig 9). From this analysis, it was very clear that the C3 cultivar was quite different from the G4 cultivar, even before exposure to conditions of drought, while the G4 (drought-sensitive) cultivar exhibited a greater change after exposure to drought.

### Prediction of transcription factors (TFs)

**Fig 10:**
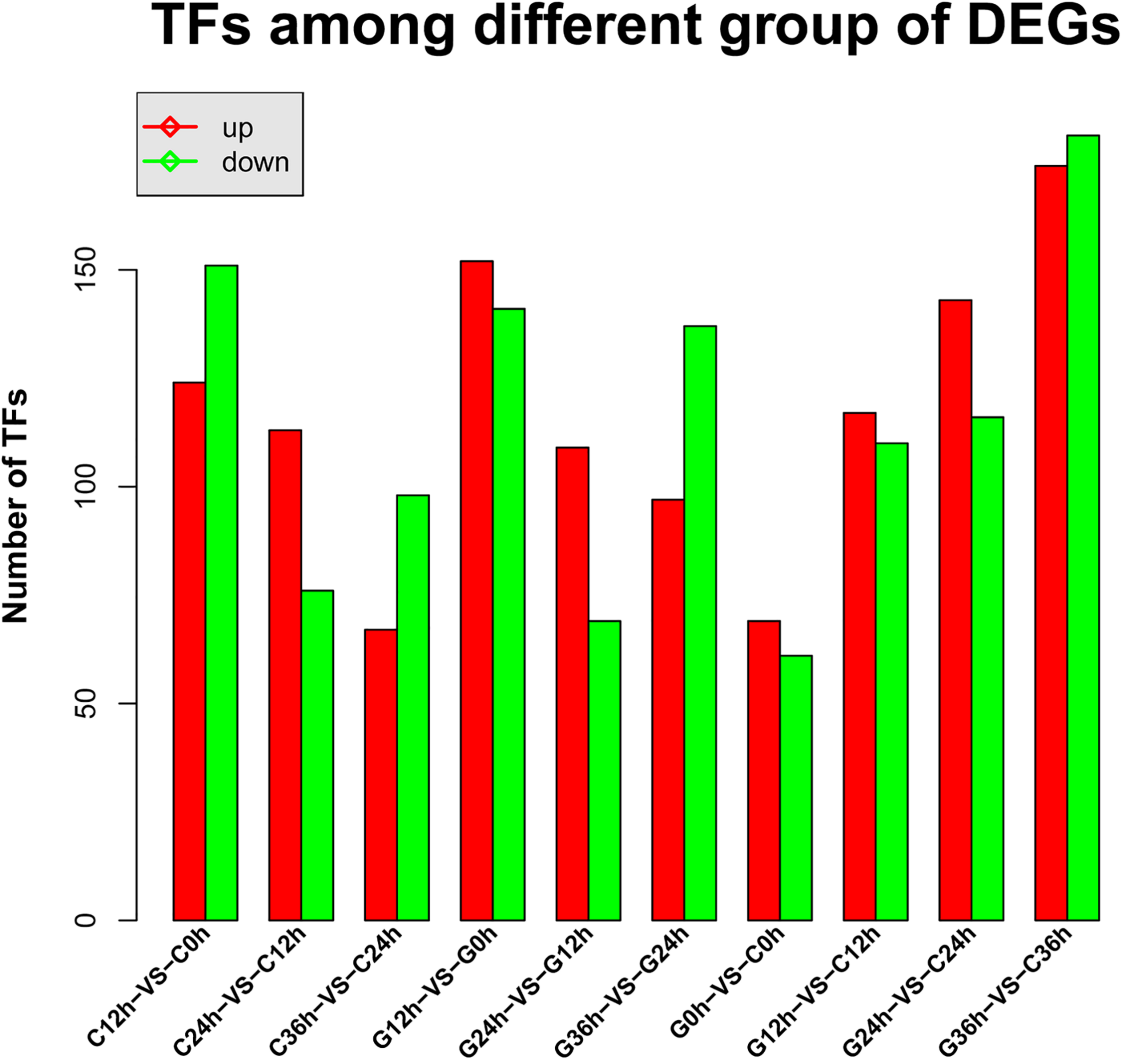
Transcription factors (TFs) identified in *Camellia oleifera*.

A total of 1,209 unigenes were identified to be involved in transcription, including 789 DEGs (Fig 10). The largest gene family was the C2H2 family, followed by the MYB-related family, bHLH family, C3H family, GRAS family, NAC family, and the WRKY family of transcription factors.

We compared the number of differentially expressed TFs between the different groups (Fig 10). In both cultivars, the number of differentially expressed TFs was the largest when comparing 0 h to 12 h of drought treatment for both up-regulated and down-regulated TFs. The C3 cultivar had 124 up-regulated and 151 down-regulated TFs, while the G4 cultivar had 152 up-regulated and 141 down-regulated TFs after 12 hours of exposure to drought stress. After 36 hours exposure to drought stress, the number of differentially expressed TFs increased dramatically in the G4 (drought sensitive) cultivar, while only increased slightly in the C3 (drought tolerant) cultivar.

Prior to drought stress, the G4 cultivar had 69 up-regulated and 61 down-regulated TFs in comparison to C3 cultivar. As the duration of drought stress continued, the number of differentially expressed TFs increased continuously.

### Physiological and biochemical indexes during drought stress

To compare the drought resistance of the two cultivars, we also detected physiological and biochemical changes during the drought stress, including the relative water content (RWC), relative electrolytic leakage (REL), chlorophyll content (Chl), peroxidase (POD) activity, malonaldehyde (MDA) content, and soluble sugar content.

**Fig 11:**
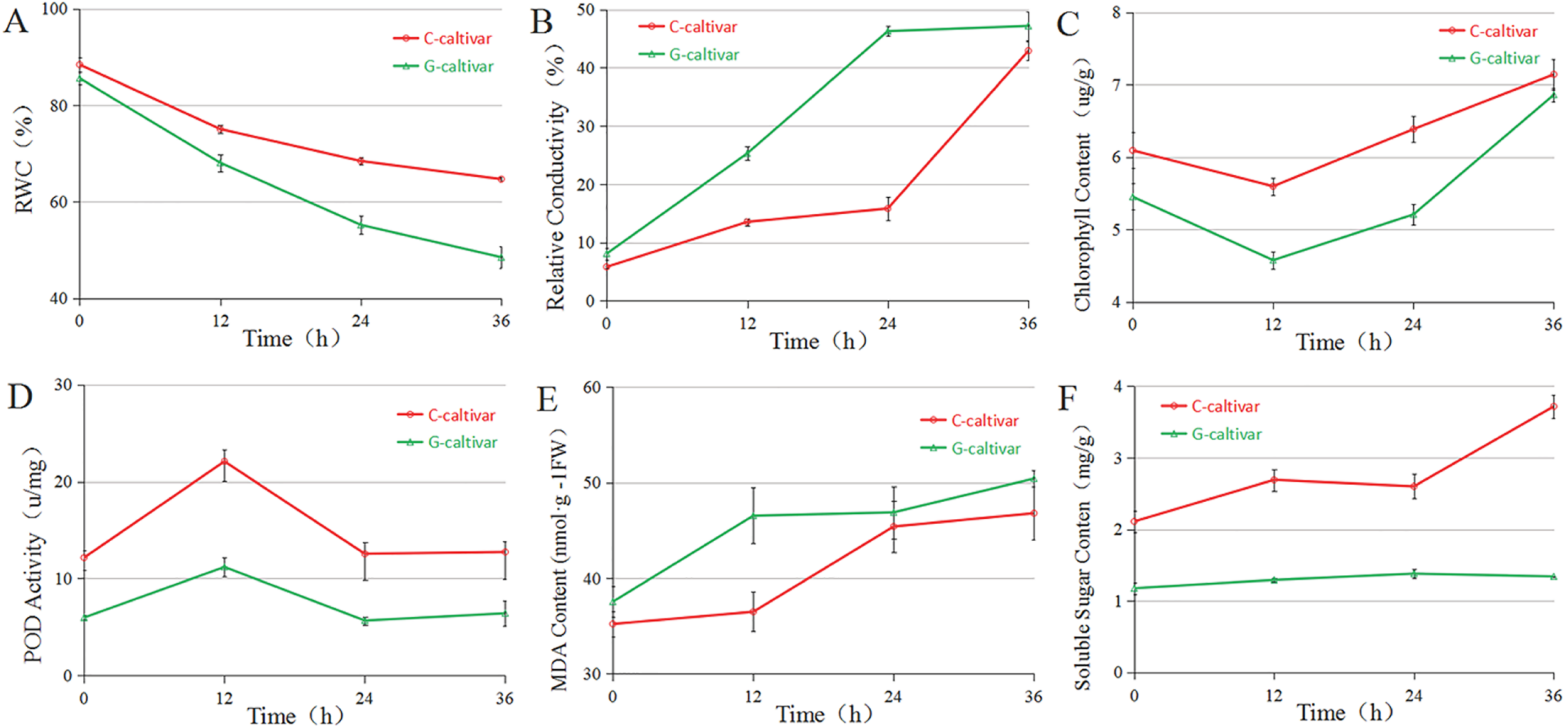
Physiological changes in the leaves of two *Camellia oleifera* cultivars in response conditions of drought. (A) Relative water content; (B) Relative conductivity; (C) Chlorophyll content; (D) Peroxidase activity; (E) MDA content; and (F) Soluble sugar content, were investigated under 20% PEG at four time points (0, 12, 24, and 36 h).

All six of the critical physiological and biochemical indexes exhibited significant changes after the drought treatment (Fig 11). In addition, the six indexes exhibited significant differences between the two cultivars during exposure to drought stress. The REL and MDA contents of the C3 cultivar were lower than those of the G4 cultivar. However, the RWC, Chl content, POD activity, and soluble sugar content were all higher in C3 cultivar.

Prior to drought stress, the relative water content (RWC) of the two cultivars was nearly identical, whereupon it declined drastically under conditions of drought. The decrease in RWC was more obvious in G4 than it was in C3. After 36 h of drought exposure, the RWC was only 56.83% in the G4 (drought-sensitive) cultivar compared to 73.13% in C3. Both cultivars displayed much higher levels of REL than control cultivars. In the G4 cultivar, REL increased rapidly after exposure to drought stress, and increased to 46.29% (after 24 h) from 8.07% (0 h), while it increased to 15.83% from 5.79% during this period in C3 cultivar, respectively.

The chlorophyll contents in the two *C*. *oleifera* cultivars exhibited interesting trends during drought stress (i.e., first decreasing and then gradually increasing). The lowest amount of chlorophyll appeared in plants exposed to drought conditions for 12 h, while the chlorophyll contents in the C3 cultivar were higher than those of G4 for the duration of the treatment. On the contrary, the POD activity of the two *C*. *oleifera* cultivars showed a tendency to increase at the beginning of the experiment, and then decrease over time. POD activity increased significantly to its highest levels at 12 h, decreased rapidly after 24 h, and then remained stable.

The MDA content increased dramatically under conditions of drought stress. In the G4 cultivar, MDA levels increased rapidly upon exposure to drought stress, from 37.55 nmol·g^−1^FW to 46.54 nmol·g^−1^FW. On the other hand, the C3 cultivar did not exhibit a significant increase until exposure to the stress for 12 h. The content of soluble sugars showed no obvious changes during drought stress in the G4 cultivar, while it increased drastically upon exposure to drought stress.

### Confirmation of gene expression by quantitative real-time PCR (qRT-PCR) analysis

Quantitative real-time PCR (qRT-PCR) was carried out to confirm the results of the genome-wide expression analysis using TaqMan probes. For this experiment, we selected 20 unigenes, all of which were differentially expressed between the two cultivars during exposure to drought stress. In confirmation of our results, all 20 unigenes selected exhibited similar expression patterns to those observed in high-throughput sequencing, proving that the high-throughput sequencing results are accurate in this study (S4 Table).

## Discussion

As sessile organisms, plants respond and adapt to stress by changes in physical structure, physiological changes, and alterations in gene expressions. These changes can maintain the balance of the synthesis of matter and energy metabolism, and can also improve a plant’s ability to survive in an arid environment. Changes in physical structure change include changes in lipid composition and stomatal conductance, and the physiological changes are mainly characterized by an increase in osmoprotectants and the synthesis of antioxidants. Plants alter their gene expression patterns when they are subjected to adverse environments[50]. The expression of functional genes can be controlled by regulatory proteins in many signal transduction pathways. For example, various protein kinases, including RLK, CDPK, MAPK, and SnPK, are important regulatory proteins in the regulation of some stress-inducible genes[51–54]. Some research has shown that protein kinases could regulate downstream functional genes when plants encounter stress.

Here, we used PEG to simulate conditions of drought. Under the stress of 20% PEG, the six physiological indexes of drought resistance were significantly changed in two *C*. *oleifera* cultivars, indicating that the drought stress stimulated the physiological and biochemical changes in these cultivars.

### Genes and transcription factors related to drought stress

We then focused on changes in the expression levels of genes associated with transcription factors. A total of 1,209 unigenes were predicted to be related to transcription, where 789 of these genes were identified as DEGs. In the C3 cultivar, 124, 113, and 67 genes were up-regulated when plants were stressed for 12, 24, and 36 h, while in the G4 cultivar, genes were152, 109, and 97 genes were up-regulated in response to drought stress, respectively.

Xia *et al*.[2] sequenced tissues of *C*. *Oleifera* using the 454 GS-FLX platform (Roche, IN, USA)and produced 84,162 unigenes, slightly more than were generated in our study (76,585). However, while there were 17,708 unigenes longer than 1,000 bp generated in the present study, Xia *et al*. detected only 2,101[2]. This difference is likely the result of a variety of factors, such as read length, the software used in assembly, and the removal of redundancy. Different software platforms often yield different results owing to variations in algorithms and parameters.

We aligned 76,585 unigenes to those produced by Xia*et al*.[2] using BLAST (E-value <1e−05). A total of 52,386 unigenes were found to be homologous to sequences in Xia’s data set. Of these, 40,610 had an alignment length of longer than 100 bp, and 11,045 unigenes had a coverage of greater than 60%. We subsequently used TGICL to cluster and assemble the two categories of unigenes, and found 22,840 unigenes in our data set that could be assembled with the unigenes discovered by Xia *et al*.

### Relative water content (RWC) and chlorophyll content

Water is an essential component of living cells, as well as an important material for metabolic activities. Drought-resistant cultivars have been shown to exhibit a leaf structure which is more conducive to reducing water loss[55]. Therefore, the water retention capacity of plant leaves directly reflects capabilities of drought resistance. In the present study, the relative water content (RWC) of both *C*. *Oleifera* cultivars decreased under conditions of drought stress. However, the RWC decreased more slowly in the C3 (drought-resistant) cultivar than that in the G4 cultivar, indicating a stronger water holding capacity in the C3 cultivar under conditions of drought stress.

A serious water deficit can cause the selective loss of plasma membrane structure and function, leading to the leakage of electrolytes and some small organic molecules, resulting in the increased conductivity of tissue soak liquid[56]. Generally, drought-tolerant cultivars exhibit lower electrolyte leakage rates than non-drought-resistant cultivars, and lower levels of the relative conductivity. Here, the electrolyte leakage rate of the G4 cultivar increased rapidly after exposure to drought stress, while this measure did not show any notable increases in the C3 cultivar until 24 h of exposure to drought.

To a certain extent, the level of chlorophyll content can reflect the photosynthetic capacity of leaves. If the stability of chlorophyll content is maintained or increased under conditions of moderate drought, the plant can survive and grow under these adverse conditions[57, 58]. Our experiment shows that chlorophyll content first decreased and then increased in both the C3 and G4 cultivars during drought stress. Many studies have shown that drought stress can cause the decomposition of chlorophyll, a decrease in the water contents of leaves and the relative concentration of chlorophyll, and an increase in fresh weight per unit of measure, which may be related to compensation and overcompensation in the face of environmental factors[59, 60]. In our study, the C3 cultivar exhibited a higher chlorophyll content than the G4 cultivar after different periods of exposure to drought stress, indicating that the C3 cultivar had a stronger photosynthetic capacity under these conditions.

### Lipoxygenase activity and MDA content

Lipoxygenase (LOX) is located in the cytoplasm, microsomes, protoplasts, and oil bodies. LOX catalyzes the lipid peroxidation of unsaturated fatty acids in membrane lipids, causing damage to the molecular structure of the membrane lipid. MDA is a highly active lipid peroxide that can cross-link lipids, sugars, nucleic acids, and proteins, and can react strongly with various components within the cell. Through its impact on cell membrane permeability and membrane proteins, MDA can influence the uptake and accumulation of ions in cells, and can affect the balance of active oxygen metabolism system[61, 62]. Under conditions of drought stress, MDA levels increased slowly in a drought-resistant cultivar while increased quickly in a drought sensitive cultivar[63]. The results of our physiological analyses showed that MDA content in the leaves of the G4 cultivar increased rapidly upon exposure to drought stress, indicating that the damage to cell membranes in the G4 cultivar was more serious in comparison to the C3 cultivar. The MDA content of the leaves of the C3 cultivar did not notably increase until it had been exposed to drought conditions for 24 h, indicating that C3 cultivar could maintain cells structure in the face of drought stress. The results of transcriptome sequencing in the present study yielded that 39 unigenes were annotated as being related to lipoxygenase (S5 Table), while 14 of these were annotated as being related to LOX activity. Of these, CL6400Contig1 exhibited no significant changes in the C3 cultivar, while it increased drastically in the G4 cultivar during exposure to drought.

### Abscisic acid (ABA)

Abscisic acid (ABA) plays an important role in the development of plants, as well as in adaptation to environmental stresses, such as cold and drought. ABA can promote stomatal closure and reduce transpiration, and thus is advantageous for plants adapting to drought. Under drought stress, the exogenous application of ABA alone, or a rapid increase of endogenous ABA content, could improve the drought resistance of plants. The type 2C protein phosphatases (PP2Cs) are critical in ABA signal transduction by negatively regulating ABA responses. PP2C can keep SnRK2 kinases—central for the ABA signaling[64]—in an inactive state in the absence of ABA. We identified eight unigenes (contig_67919, CL1390Contig2, contig_42750, contig_2266, contig_65705, contig_18712, and contig_107511) related to PP2C. Of these, contig_67919 and contig_42750 were significantly down-regulated during exposure to drought stress. Moreover, we also identified CL10598Contig1 to be related to SnRK2, which showed no significant increases in the C3 cultivar, while being significantly up-regulated in the G4 cultivar during drought stress.

In higher plants, ABA is synthesized by 9-cis-epoxycarotenoid dioxygenase (NCED) from violaxanthin. In our study, four unigenes (contig_71401, CL11858Contig1, CL8035Contig1, and contig_101053) were identified to be related to NCED. Contig_71401 and CL11858Contig1, were significantly up-regulated after 12 hours of drought stress, and contig_101053 was down-regulated in both C3 and G4 cultivars. However, CL8035Contig1 were significantly up-regulated in G4 cultivar while showed no significant increase in expression in C3 cultivar.

### Protective enzyme system

Abiotic stress causes plants to form excessive quantities of reactive oxygen species (ROS), which in turn play a strong role in the destruction of the cell membrane system, lipids, proteins, nucleic acids, and other macromolecules. To survive, plants have developed a protective enzyme system to remove excessive ROS. This protective enzyme system is an antioxidant system, which is mainly composed of superoxide dismutase (SOD), catalase (CAT), and peroxidase (POD).

In our study, 21 unigenes were associated with SOD. Of these, four unigenes (CL1731Contig1, contig_37253, contig_73481, and contig_37254) were remarkably down-regulated in the G4 cultivar in comparison to the C3 cultivar during conditions of drought stress. 52 unigenes were identified to be related to catalase (CAT), and 32 were annotated as being involved in catalase activity, where only contig_11343 was up-regulated during drought stress.

As a member of the group of free radical-scavenging enzymes, POD is closely linked to drought resistance in plants, where drought-tolerant cultivars generally have high levels of POD activity[65]. According to the results of our physiological analyses, POD activity increased to a greater extent in the C3 (drought-resistant) cultivar than it did in the G4 cultivar after12 h of drought stress. Furthermore, POD activity of the C3 cultivar was higher than that of the G4 cultivar at all stages of drought stress, indicating that the POD activity of the C3 cultivar was activated to a large degree. In our transcriptome sequencing data, 100 unigenes were associated with POD activity, where 25 of these unigenes were up-regulated in both cultivars during drought stress.

### Vitamin B6 metabolism

The metabolic systems of vitamin B6 (VB6) that are known to be involved in the stress response were differentially regulated between control and drought-exposed plants. In recent years, researchers have discovered that VB6 is an antioxidant that can effectively quench oxygen singlets and superoxide anion radicals, and can regulate cell signaling molecules and ion channels associated with cell membranes. VB6 is also known to protect plants from being affected by osmotic pressure, temperature, ultraviolet radiation, oxidation, and other environmental stresses.

**Fig 12:**
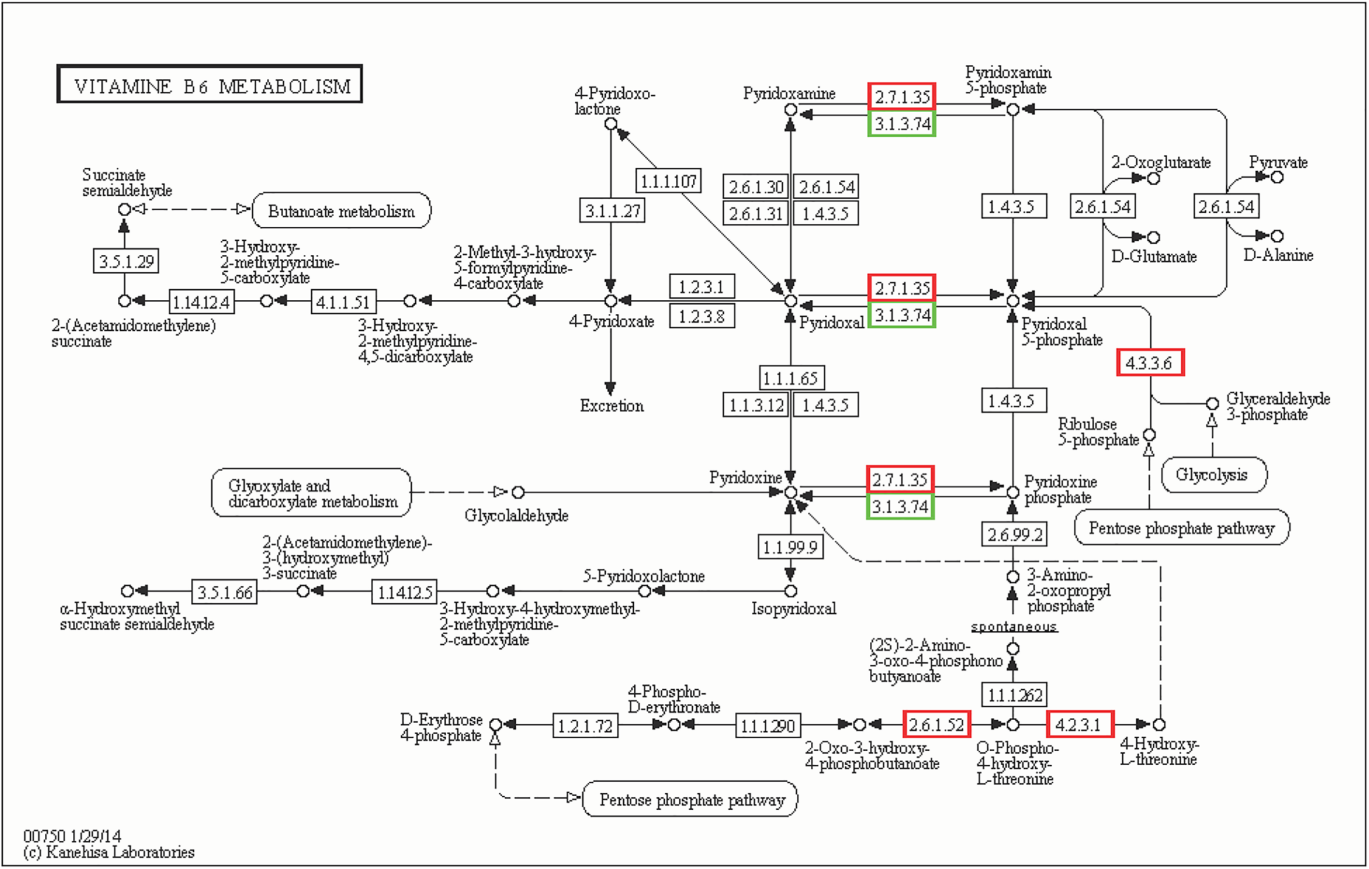
Vitamin B6 metabolism in *Camellia oleifera*.

In our study, when the C3 cultivar was exposed to conditions of drought for 12 hours, E.C.2.7.1.35, E.C.4.3.3.6, E.C.4.2.3.1, and E.C.2.6.1.52 were up-regulated, while E.C.3.1.3.74 was down-regulated. E.C.2.7.1.35 and E.C.3.1.3.74 were annotated as being pyridoxal kinase and pyridoxal phosphatase, respectively (Fig 12).

### Osmolytes

Plants adapt to drought stress by accumulating osmolytes under low water potential, proline (Pro) being one of the most important osmolytes. P5C reductase (P5CR) is a critical enzyme in the biosynthesis of proline in higher plants[66]. In our study, contig_29559 was identified to be related to P5CR, and exhibited a sharp increase in expression after 12 hours of drought stress in both cultivars. After 12 h, the expression of P5CR decreased, where P5CR expression declined more rapidly in theG4 cultivar. Another important enzyme in proline biosynthesis is delta(1)-pyrroline-5-carboxylate synthase (P5CS). In our study, there were five unigenes (CL10313Contig1, contig_37315, contig_57036, contig_23733, contig_43412) related to P5CS, although none of them showed variations in expression profiles during exposure to drought stress.

Another important osmolyte is betaine, which can also reduce water potential under conditions of drought stress. We identified two unigenes (CL4202Contig1, contig_32576) that are related to betaine-aldehyde dehydrogenase, which produces glycine betaine by oxidizing betaine aldehyde. The expression of CL4202Contig1 increased drastically in both cultivars at 12 h of drought stress. However, contig_32576 was down-regulated after 12 hours and the fold-change in the C3 cultivar was much smaller than that of the G4 cultivar.

Soluble sugar, an important substance in plants for osmotic adjustments, can aid cells in preventing dehydration, and is conducive to drought-resistance[67]. Under drought stress, the soluble sugar content in the leaves of the C3 cultivar increased significantly when stressed for 12 and 36 h and maintained a high level over the course of exposure to drought stress, while exhibiting no notable increase in the leaves of the G4 cultivar. It is likely that by increasing the soluble sugar content, the C3 cultivar increased the anti-dehydration ability of cells, thereby slowing the damage caused by drought, while the capacity for osmotic adjustment in the G4 cultivar is virtually non-existent. According to the functional annotations in the present study, 18 unigenes were identified to be related to sucrose synthase (SS), a class of enzymes essential for the biosynthesis of sucrose. Only one unigene (CL11509Contig1) showed significant changes in both cultivars during exposure to drought stress. In both cultivars, CL11509Contig1 was down-regulated after 12 h of drought, was significantly up-regulated after 24 h, and was then down-regulated again after 36 h.

### Secondary metabolites

Under drought stress, the concentrations of secondary metabolites often increases in plant tissues, including terpenoids, alkaloids, and organic acids. Although the functions of some secondary metabolites are not yet clear, the correlation between quantity and drought resistance indicate they play a role in physiological responses to drought. In our study, 28 unigenes were found to be related to the biosynthesis of sesquiterpenoid and triterpenoid (S6 Table). After being treated with 20% PEG for 12 hours, 15 and 13 unigenes were significantly enriched in the sesquiterpenoid and triterpenoid biosynthesis pathway, and were down-regulated in the C3 and G4 cultivars, respectively. No unigenes related to this pathway were found to be up-regulated.

**Fig 13:**
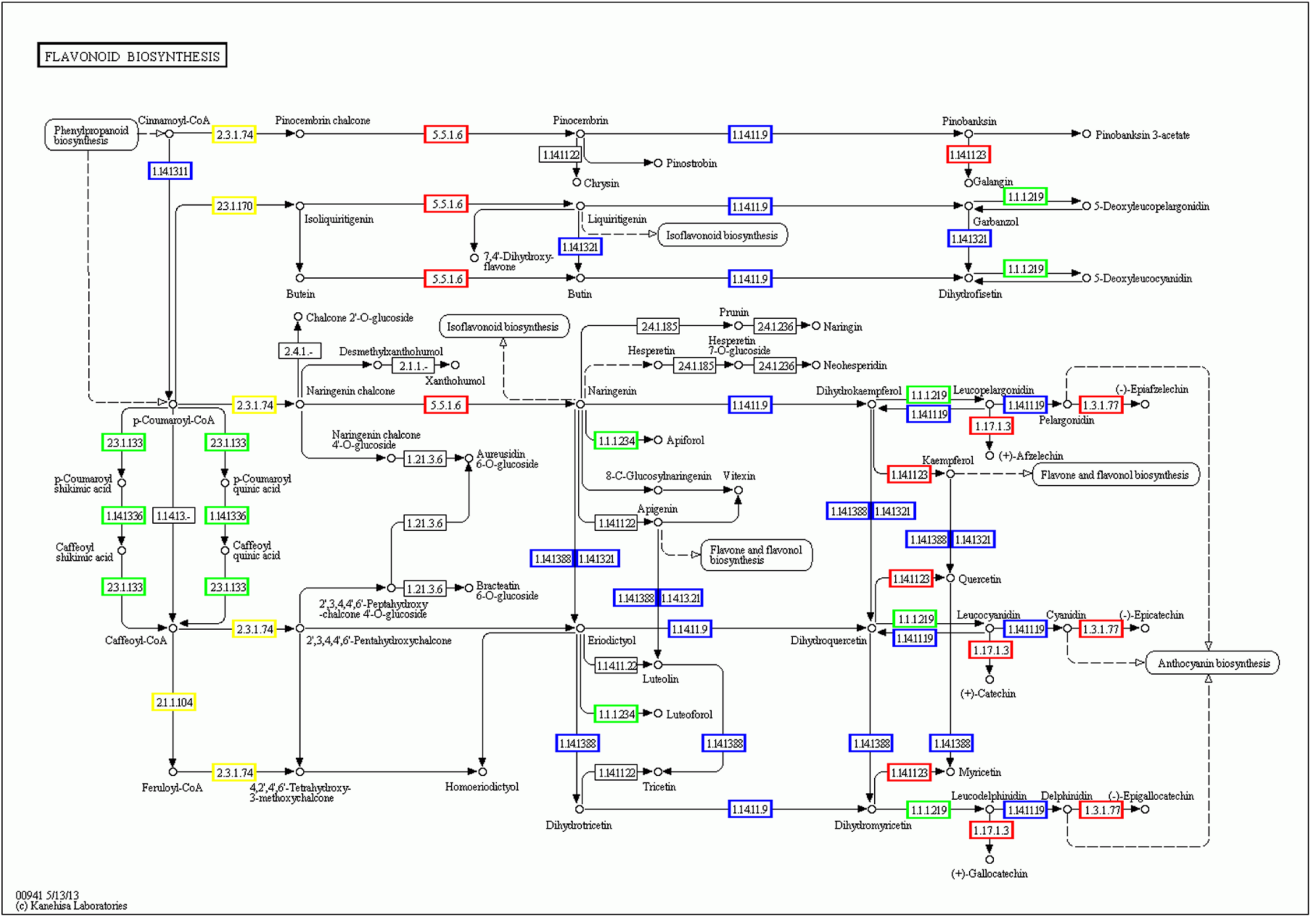
Flavonoid biosynthesis in *Camellia oleifera*.

Flavonoids are a major class of plant secondary metabolites that play an important role in many functions, such as in pigments and antioxidant activity. Studies have shown that under conditions of abiotic stress, the overexpression of synthetic flavonol glycosides and anthocyanins in plants can effectively remove reactive oxygen species, thereby enhancing the drought tolerance of plants[68,69]. We found 60 unigenes related to flavonoid biosynthesis in the present study (Fig 13). When exposed to conditions of drought for 12 h, 13 and 11 unigenes were found to be down-regulated in the C3 and G4 cultivars, respectively, while 9 and 5 unigenes were up-regulated in the C3 and G4 cultivars, respectively.

## Materials and methods

### Plant materials and dehydration treatment

One-year-old seedlings of two *C*.*oleifera* cultivars—drought-tolerant cultivar ‘Cenruan No.3’ (C3) and drought-sensitive cultivar ‘Guiwu No.4’ (G4)—were used and verified in this study. All seedlings were cultivated at the Guangdong State Farms Tropical Crop Research Institute.

The treatment for both C3 cultivar and G4 cultivar groups were carried out in an artificial climate chamber for three months under the following conditions: 150 μmol·m^−2^·s^−1^ (fluorescent tubes), photoperiod 14 h light/10 h darkness, and a temperature of 25±2°C. First, seedlings were transported under the same conditions as the climate chamber for one week. Then, the seedling plants were separated from pots, the soil washed with deionized water, and were cultivated in half-strength Hoagland’s solution with insufflating air for two days to accommodate the liquid environment before drought treatments. After a smooth adaptation period, drought treatment was applied to two *C*. *oleifera* cultivars. 20 seedlings of each cultivar were subjected to drought stress using a 20% PEG-6000 nutrient solution (a non-permeant osmolyte widly used in experiments to mimic dehydration stress), as the drought treatment with the symptoms of wilting for 12 hours, 24 hours, and 36 hours; while a second set of the two cultivar seedlings were used as a control in the absence of PEG-6000. For each treatment and control, leaves were collected from 5 seedlings, mixed together, kept in liquid nitrogen and stored at −80°C for RNA extraction.

### Measurement of physiological parameters

All samples were immediately assessed using physiology indexes: the relative water content (RWC) was detected using the saturated water method; the relative electric conductivity (REL) was detected using the electrical conductivity method; chlorophyll content was detected using the electrical conductivity method; the activities of peroxidase (POD) were detected via a guaiacol colorimetric assay. malondialdehyde (MDA) content was detected using thiobarbituric acid chromatometry; soluble sugar content was detected using an anthrone colorimetric assay.

### RNA extraction and determination of quality

Total RNA was extracted three times using a CTAB procedure as previously described[70, 71], and were finally dissolved in 10 mol·L^−1^Tris-HCl buffer (PH 7.6). The purity of the RNA samples according to the A260/A280 ratio was determined using the NanoDrop ND-8000; the A260/A280 ratios of all samples were in the approximate range of 1.9–2.1. The integrity of the RNA samples was assessed with an Agilent 2100 Bioanalyzer, and samples with no sign of degradation were selected for further analysis.

### cDNA library construction and sequencing

The cDNA library was constructed using the NEBNext® Ultra^TM^ RNA kit according to the manufacturer’s instructions. In brief, 45 μg of total RNA from each sample was prepared at a concentration of approximately 1,500 ng·μL^−1^. Poly (A) mRNAs were enriched using oligo (dT) beads and interrupted into short fragments with fragmentation buffer. First-strand cDNA was synthesized using hexamer primers and reverse transcriptase (Invitrogen). The second-strand cDNA was then synthesized using buffer, dNTPs, RNaseH (Invitrogen), and DNA polymerase I (New England BioLabs).

For the construction of the two paired-end libraries, the double-stranded fragments were then purified using a QiaQuick PCR extraction kit and resolved with EB buffer to finish the end reparation, and were connected using sequencing adaptors. The successfully connected fragments were then subjected to sequencing using the Illumina HiSeqTM 2500 sequencing platform. The raw reads were initially processed by removing adaptor sequences and low quality reads using an in-house Perl software, where the average proportion of clean reads in each sample was approximately 91.5%. the low quality reads here refers to reads whose ratios of unknown bases are higher than 5%, or ratios of low quality bases (base quality≤10) are higher than 20%.

### *De novo* assembly and assessment

*De novo* transcriptome assembly was performed using the short-reads assembly program, Clc_assembler (https://www.qiagenbioinformatics.com/products/clc-assembly-cell/), which first combined reads with a certain degree of sequence overlap into contigs for each sample, Secondly, we used TGICL (http://www.mybiosoftware.com/tgicl-2-1-tgi-clustering-tools.html), a software platform for the rapid clustering of large EST datasets, to assemble all the unigenes from all samples to form a single set of non-redundant unigenes.

After clustering, the unigenes were divided into two classes: clusters and singletons. Finally, a BLASTx alignment (E-value < 0.00001) was performed between unigenes and protein databases, with the following priority order: non-redundant (nr; NBCI), SwissProt (ExPASy), KEGG (http://www.genome.jp/kegg/kegg1.html), and COG (ftp://ftp.ncbi.nlm.nih.gov/pub/COG/). The sequencing direction of unigenes was decided by the best alignment results. For unigenes that could not be aligned to any of the above databases, we used ESTScan (https://sourceforge.net/projects/estscan/) to determine the sequencing direction.

### Functional annotation

Unigene sequences were aligned to the aforementioned protein databases using BLASTx (E-value < 0.00001) and to the nucleotide sequence database ‘nt’ (E-value < 0.00001) using BLASTn. We selected proteins with the highest similarity for the corresponding functional annotations of the given unigenes.

Based on the nr annotation, the GO (Gene Ontology) functional annotation was obtained using the Balst2GO program. In order to understand the distribution of gene functions on the macro level, we also used WEGO software (http://wego.genomics.org.cn/cgi-bin/wego/index.pl) to classify the GO functions. The COG functional annotations were obtained by aligning the unigenes to the COG database. Next, we further examined the potential complex biological networks of the unigenes by aligning them to the KEGG database in order to obtain metabolic pathway connections.

### Protein-coding region prediction

We aligned unigenes to the aforementioned protein databases. Based on the BLAST results, the coding regions of unigenes were determined by proteins with the highest similarity. Subsequently, we translated the coding region sequences into amino acid sequences according to the standard codon table. In this way, we acquired both the amino acid and nucleotide (5'–3') sequences of the coding regions of unigenes.

### Differential expression analysis of unigenes

The expression levels of the unigenes were calculated by using the FPKM (fragments per kb per million fragments) method, according to the following formula:

$$FPKM=\frac{C*10^{6}}{NL/10^{3}}$$

where C is the number of fragments which were uniquely mapped to a given unigene, N is the total number of fragments that were uniquely mapped to all unigenes, and L is the length (in kb) of a given unigene.

The statistical significance of the differential expression profile for each gene was determined according to the method described by Audic and Claverie[72]. The FDR (False Discovery Rate) control method was used in the testing of multiple hypotheses to correct the results for p value. Unigenes with an FDR of <0.001 and a log2 ratio of the absolute value ≥ 1were considered to be a differentially expressed genes (DEG).

We then mapped all DEGs to each term in the Gene Ontology (GO) database (http://www.geneontology.org/), calculated the gene numbers in each GO term, and obtained a gene list and number of genes for every GO term. Using a hypergeometric test, we found significantly enriched GO terms in DEGs in comparison to the genomic background of *C*. *oleifera*. Calculated *p* values were subjected to a Bonferroni Correction, yielding a corrected *p* value ≤ 0.05 as the threshold. GO terms fulfilling this condition were defined as significantly enriched GO terms in DEGs. The *p* value was estimated according to the following formula:

P=1−∑i=0m−1(Mi)(N−Mn−i)(Nn)

where N is the number of all genes with a GO annotation, n is the number of DEGs in N, M is the number of all genes that are annotated to the certain GO terms, and m is the number of DEGs in M.

DEGs were also subjected to pathway enrichment analysis. We calculated the gene numbers in each pathway by mapping all DEGs to the KEGG database (http://www.genome.jp/kegg). By comparing significantly enriched GO terms in DEGs with the whole genome background of *C*. *oleifera*, pathways with p ≤ 0.05 were designated as significantly enriched in DEGs using the same multiple testing correction method as GO enrichment analysis. Using pathway enrichment analysis, we were able to obtain the main biochemical pathways and signal transduction pathways in which the DEGs were involved, including some pathways related to drought stress.

### Quantitative real-time PCR (qRT-PCR) analysis

Real-time quantitative PCR analyses were performed on selected unigenes. The first-strand cDNA was synthesized using SuperScript® III Reverse Transcriptase (ThermoFisher, USA). The real-time quantitative PCR reaction was performed on a ABI 7500 Real-Time PCR System (Applied Biosystems, USA) using PLATINUM TAQ DNA POLYMERASE (ThermoFisher, USA). The reaction mixture (20 μL) was comprised of 2 μL of 10 × PCR buffer/20 × SYBR solution, 0.5 μL of each primer (10 μmol/L), and 1μL of cDNA template. Relative gene expression levels were calculated using the 2^−ΔCt^. The GAPDH gene was used as the internal control. No-template controls and melting curve analyses were included for each gene during each run. The primer sequences used for qRT-PCR are listed in S4 Table.

## Conclusions

This is the first report of transcriptome sequencing for *C*. *oleifera* exposed to conditions of drought. The total length of the reads was ~11.9 Gb. A total of 76,585 unigenes were assembled, 52,531 (68.6%) of which were functionally annotated. Using enrichment analyses of the DEGs against the GO and KEGG databases, we identified DEGs which were significantly enriched in pathways involved in the biosynthesis of osmolytes, secondary metabolites, and in the regulation of the activity of protective enzymes. The large number of transcriptomic sequences and their functional annotations elucidated in the present study provide sufficient resources for further molecular studies of *C*.*oleifera*. Moreover, information on the KEGG metabolic pathways and the differential expression of transcription factors will facilitate the discovery of other drought-resistant genes in this commercially valuable species.

## Supporting information

S1 Fig Length distribution of contigs. (TIF)

S2 Fig Histogram of protein length. (TIF)

S1 Table DEGs. Differentially expressed unigenes.
(XLSX)

S2 Table GO enrichment. GO enrichment analyses of the DEGs.
(XLSX)

S3 Table KEGG enrichment. KEGG enrichment analyses of the DEGs.
(XLSX)

S4 Table RT-PCRvalidation. Validation of 20 unigenes.
(XLS)

S5 Table Lipoxygenase. Unigenes related to lipoxygenase.
(XLS)

S6 Table Secondary metabolites. Unigenes related tosecondary metabolites.
(XLS)
