# Supplementary figures and images for: Transcriptome analysis of the tea oil camellia (Camellia oleifera) reveals candidate drought stress genes
Source: PLoS One. 2017 Jul 31;12(7):e0181835. doi: 10.1371/journal.pone.0181835 (PMC5536306; doi:10.1371/journal.pone.0181835)

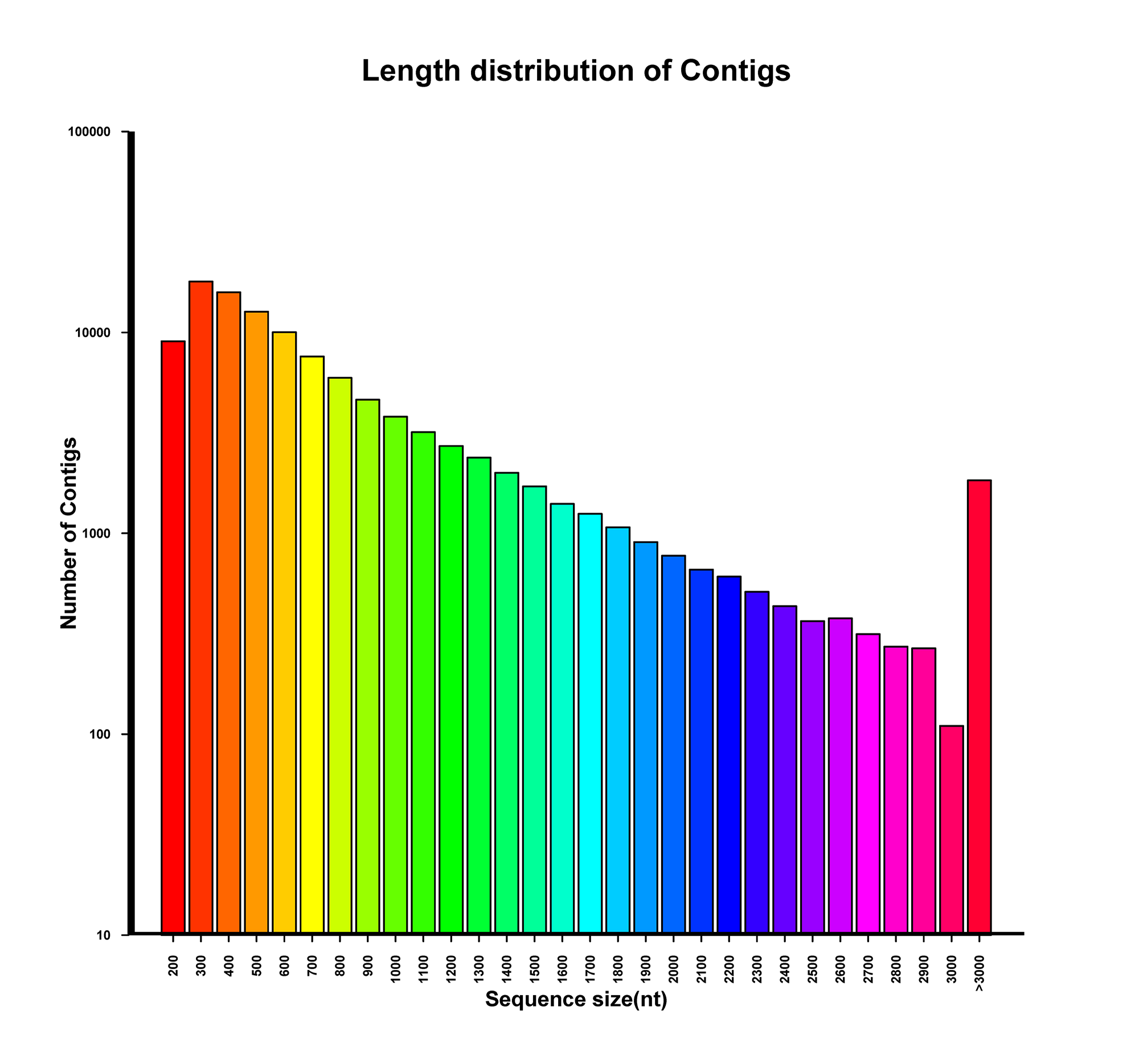

Supplement: S1 Fig — (TIF) [file pone.0181835.s001.tif]
